# Visceral Artery Aneurysms in the Presence of Upstream Stenoses

**DOI:** 10.3390/jcm13113170

**Published:** 2024-05-28

**Authors:** Amun Hofmann, Philipp Schuch, Franz Berger, Fadi Taher, Afshin Assadian

**Affiliations:** 1 Department of Vascular and Endovascular Surgery, Klinik Ottakring, 1160 Vienna, Austria; 2 Department of General Surgery, Klinik Ottakring, 1160 Vienna, Austria

**Keywords:** Sutton–Kadir syndrome, median arcuate ligament, visceral artery aneurysm

## Abstract

**Background**: Sutton–Kadir syndrome describes a rare pathology that commonly includes an aneurysm of the inferior pancreaticoduodenal artery in combination with a celiac trunk stenosis or occlusion, often caused by median arcuate ligament compression. Several therapeutic approaches exist including open surgical, endovascular, and hybrid treatments. Other combinations of visceral artery aneurysms and upstream stenoses exist but the cumulative body of evidence on these combinations is weak due to their rarity. **Methods**: A retrospective analysis of patient data from a single center was carried out. Electronic patient records were filtered for keywords including “visceral aneurysm”, “Sutton–Kadir”, and “median arcuate ligament”. Imaging studies were re-examined by two blinded vascular surgeons with a third vascular surgeon as a referee in case of diverging results. **Results**: Sixteen patients had a visceral artery aneurysm with an upstream stenosis. All cases had a celiac trunk obstruction while one patient also had a concomitant superior mesenteric artery stenosis. Both median arcuate ligament compression and atherosclerotic lesions were identified. The location of the aneurysms varied even though the inferior pancreaticoduodenal artery was most frequently affected. A classification system based on the different combinations of stenoses and aneurysms is presented and introduced as a new pathologic entity: visceral artery aneurysm in the presence of upstream stenosis (VAPUS). **Conclusions**: The concomitant presence of visceral artery aneurysms, especially in the pancreaticoduodenal arteries, and blood flow impairment of the celiac axis or superior mesenteric artery is a rare pathology. The proposed VAPUS classification system offers an accessible and transparent route to the precise localization of the affected vessels.

## 1. Introduction

When Sutton and Lawton published their work on celiac artery stenoses or occlusions with concomitant aneurysms of the collateral supply in 1973 [1] the pathophysiology was not conclusively understood. Five years later in 1978, Kadir and colleagues published a case series of four patients with aneurysms of the inferior pancreaticoduodenal arteries (IPDA) in association with celiac axis occlusions [2]; they not only showed that this finding constitutes a pathological entity but also postulated a mechanical pathway for it. Since then, IPDA aneurysms in association with stenosis or occlusion of the celiac trunk have been frequently termed Sutton–Kadir syndrome. It has been repeatedly discussed that the increased retrograde blood flow through the pancreaticoduodenal arcades induces outward remodeling to accommodate the increased volume that subsequently turns into aneurysmatic enlargement [2,3,4,5]. However, IPDA aneurysms might also develop in the presence of stenoses downstream from the celiac axis at the common hepatic artery [6]. Even though the compensatory vessel enlargement hypothesis has been repeatedly discussed as the culprit for these aneurysms, the pathomechanism has not been conclusively solved so far.

It has been reported that, in approximately 20% of cases, impaired blood flow through the celiac artery is caused by compression of the median arcuate ligament (MALC) of the diaphragm [7], which, in the presence of clinical symptoms, is often referred to as Dunbar syndrome [8]. Apart from external compression, atherosclerotic lesions of the celiac trunk have also been reported in the presence of peri-pancreatic aneurysms [9]. Nevertheless, IPDA aneurysms that are secondary to superior mesenteric artery (SMA) obstructions [10,11] or in the absence of celiac axis impairments [12] have also been reported. Additionally, aneurysmatic degeneration might affect the other peripancreatic arteries, specifically the gastroduodenal or superior pancreaticoduodenal arteries, instead of the IPDA, thereby further expanding the heterogeneity of potential presentations [11].

Visceral aneurysms in general and those of the pancreaticoduodenal arteries in specific are believed to be at an elevated risk for rupture even at small diameters [13], though the underlying reasons have not yet been conclusively investigated. Therefore, prophylactic treatment is commonly warranted. However, currently no superior treatment approach exists that has been continuously proved to optimize long-term outcomes, which might be attributed to the very low incidence of the pathology. Both surgical and interventional therapy have been reported in the literature as well as a hybrid combination of the two [4]. The European Society for Vascular Surgery’s guidelines on mesenteric vessels diseases recommend arterial reconstruction for mesenteric aneurysms when technically feasible in non-high risk surgical candidates [14], while the Society for Vascular Surgery primarily suggests endovascular aneurysm treatment [15]. Endovascular treatment commonly involves transcatheter embolization of the aneurysm and has satisfying technical and clinical success rates [16,17]. Case reports have also proved the feasibility of computed tomography- [18] and ultrasound-guided [19] percutaneous transabdominal thrombin injections. It is frequently suggested that celiac artery stenosis should undergo concurrent treatment, which in case of endovascular treatment strategies can be achieved by celiac artery stenting [20]. Various surgical approaches have been reported including ligation, resection, or endoaneurysmorraphy which are commonly combined with a division of the median arcuate ligament to treat the upstream stenosis or occlusion [7]. Excision of the aneurysm might also be combined with an aorto-hepatic bypass [21]. Both surgical and endovascular approaches are reported to produce good results even though follow-up periods might vary significantly in length [22].

The present work features a retrospective analysis of cases with a visceral artery aneurysm and an additional upstream stenosis that received treatment at a vascular surgery center. Due to the size of the study population, the analysis focuses on qualitative descriptive methods.

## 2. Methods

### 2.1. IRB Approval

The study was approved by the appropriate institutional review board and ethics committee of the City government of Vienna (ID: 23-169-VK) on 20 October 2023. Approval included a waiver of informed consent for this retrospective analysis. The study was conducted according to the Declaration of Helsinki.

### 2.2. Design

The present investigation is a retrospective single-center cohort study. 

### 2.3. Data

Patients were identified by search queries including Sutton–Kadir, Dunbar, median arcuate ligament, or visceral artery aneurysm in the electronic patient documentation. All patient-specific information such as comorbidities, surgery reports, or follow-up documentation were extracted from the digital health records. Data from imaging investigations into such as affected vessels or aneurysm diameters were obtained by two board certified vascular surgeons who re-analyzed images blinded to each other. Data were then merged, checked for discrepancies, and diameter means were calculated. The two examiners identified the identical arteries and maximum diameters measurements were all within a 25% difference. In 3 patients, occlusion etiology diverged between the raters and in 2 patients the aneurysmatic vessel was labelled differently. A third vascular surgeon re-examined the respective imaging studies to reach a final conclusion. 

### 2.4. Analysis

Patient characteristics were analyzed by descriptive statistical methods including calculation of measure of central tendency and dispersion. All statistical analyses were performed with R version 4.1.3 (R Foundation for Statistical Computing, Vienna, Austria) in RStudio (Posit PBC, Boston, MA, USA).

## 3. Results

### 3.1. Sample Characteristics

Between January 2014 and January 2024, 56 patients underwent consultations at the outpatient clinic for either median arcuate ligament syndromes or prevalent visceral artery aneurysms. Sixteen patients (28.6%) were identified with a documented concomitant visceral artery aneurysm and upstream stenosis. In summary, 14 patients underwent surgery and/or endovascular intervention, of which 13 survived the peri-operative period (30 days). The median follow-up time was 3.2 years. Only three patients in our cohort were treated due to an acute rupture of their aneurysm. The baseline sample characteristics are shown in Table 1.

### 3.2. Anatomic Features and New Classification

**Figure 1:**
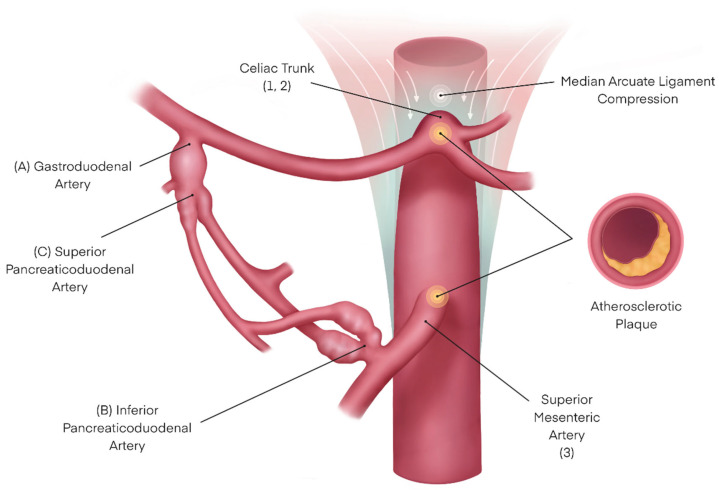
Illustration of the different pathologies that might contribute to VAPUS, including designation according to the proposed classification (in brackets).

**Figure 2:**
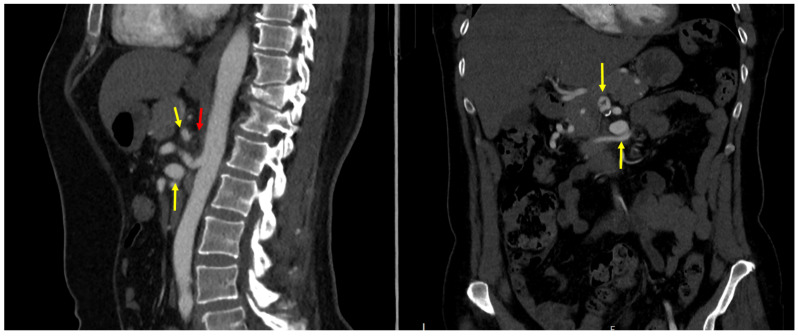
Computed tomography angiography of a VAPUS 1 A-B with a celiac axis stenosis caused by median arcuate ligament compression (red arrow) and an aneurysm of the gastroduodenal artery (upper yellow arrow) as well as the inferior pancreaticoduodenal artery (bottom yellow arrow).

The pre-operative imaging studies were re-analyzed. Based on the data collected from our patients as well as published literature, we propose the introduction of a new distinct entity: visceral artery aneurysm in the presence of upstream stenosis (VAPUS). VAPUS can be classified based on the obstructed aortic branch and its etiology (VAPUS class 1–3) as well as the aneurysmatic vessel downstream of it (VAPUS class A–C). We propose a classification that distinguishes between externally compressed (such as MALC) and endoluminally obstructed celiac arteries to depict the distinct pathophysiology of both which require different therapeutic approaches. (Table 2 as well as Figure 1 and Figure 2).

Out of all sixteen patients, nine (56.3%) had a celiac trunk stenosis with an associated aneurysm of the IPDA, corresponding to a VAPUS 1(-2) B or previous Sutton–Kadir syndrome diagnosis. All of these patients featured an MALC while one patient had a combined pathology of MALC and atherosclerotic lesions. Two patients had clinical evidence of Dunbar syndrome. Other pathologies included a combined celiac trunk and SMA obstruction with an IPDA aneurysm (VAPUS 1–3 B), or aneurysms in other visceral branches such as the gastroduodenal artery prior to the superior pancreaticoduodenal arteries (VAPUS X A), or the anterior superior pancreaticoduodenal artery (VAPUS X C). (Table 3) We decided to provisionally exclude aneurysms of the splenic artery and its branches (*n* = 3) from the classification system due to an inconclusive relationship between an aneurysm and concomitant celiac trunk stenosis.

### 3.3. Treatment Strategies

**Figure 3:**
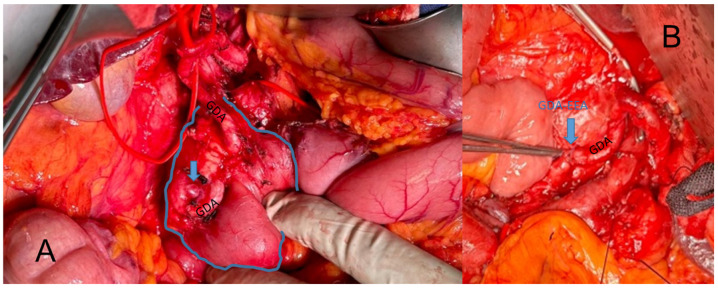
Intraoperative depiction of a VAPUS X A case with an aneurysm of the gastroduodenal artery (GDA) before (**A**) and after (**B**) resection and end-to-end anastomosis (EEA).

The aim of therapy was to first re-establish superior blood flow to the hepatic artery, and second, to resect or exclude the aneurysm either surgically or in a secondary procedure by coil embolization. Out of the fifteen patients undergoing surgery, seven (46.7%) were treated by a total aneurysm resection, of which six cases received an end-to-end anastomosis (one during a primary Whipple procedure due to pancreatic cancer, Figure 3A,B) of the unaffected sections of the original vessels while one patient received a venous interposition graft using a great saphenous vein graft. Three patients underwent aneurysm embolization in a secondary intervention, while two aneurysms were coil embolized during an acute rupture. In one case the aneurysm was simply ligated. Two patients with a secondary intervention for embolization also received a stent to treat the upstream stenosis (one in the celiac artery and one in superior mesenteric artery). Four patients were treated with a bypass surgery to facilitate revascularization downstream of the affected stenotic segment (three of them were aorto-hepatic including one secondary to embolization during rupture and one that was aorto-splenic). In 10 patients (62.5%) the concomitant MALC was treated by resection of the impairing soft and nerval tissues. Almost all patients received a multifaceted treatment addressing both the aneurysm and facilitating revascularization with just a single case undergoing aneurysm resection only. Treatment strategies are shown in Table 4.

### 3.4. Outcomes

One patient that was operated on died during the peri-operative period. The post-operative CTA showed no signs of bleeding and the most likely attributable cause was anaphylactic shock. The remaining patients received surgery between June 2014 and January 2024. Therefore, the lengths of their potential follow-up periods inherently varied. The longest follow-up period was 8.2 years after surgery. Four patients dropped out of the follow-up regimen at our clinic, while eight patients with documented outpatient clinic visits up to the present year remained free of any additional invasive intervention since their primary treatment (mid- and long-term follow-up not available for the two latest cases). Irrespective of the therapeutic approach, all treatment strategies can be considered as technical successes.

## 4. Discussion

With the present work we aimed to characterize a new distinct pathologic entity: visceral artery aneurysms in the presence of upstream stenosis—VAPUS. We argue that what was previously referred to as Sutton–Kadir syndrome, an IPDA aneurysm in the presence of a celiac artery stenosis, is the most frequent form of VAPUS, but this is not the only observed combination. Patients undergoing treatment at our center also showed different combinations of aneurysmatic and stenotic/occluded vessels reflecting the heterogeneity of the pathology and corresponding with previous reports in the literature that also feature other combinations.

In our case series, 15 patients had an obstruction of the celiac trunk or SMA combined with a visceral artery aneurysm (excluding splenic branches). Most frequently, the aneurysm affects the IPDA corresponding to previously identified Sutton–Kadir syndrome (VAPUS 1 and/or 2 B). However, other combinations exist, and the potential variety of affected vessels and underlying pathologies (MALC and atherosclerosis) necessitates a classification system that not only reflects this but also assists in precisely describing the location and etiology to define a fitting therapeutic approach. For example, obstruction caused by MALC (VAPUS 1 X) requires surgical release whereas atherosclerotic lesions in the celiac trunk and SMA (VAPUS 2 or 3 X) might be accessible through endovascular solutions.

While most of the patients in our study cohort presented with flow obstruction/aneurysm combinations that corresponded well with cases in the literature, one patient had a combined MALC and atherosclerotic stenosis of the celiac artery with an aneurysm in a splenic side branch. The patient was treated for to acute rupture after being transferred from another local hospital and the exact culprit vessel could not be accurately identified due to intra-abdominal hemorrhage. In a different patient we observed a post-stenotic dilation of the splenic artery but we would not expect this to continue into a splenic side branch resulting in aneurysmatic enlargement there, while a third patient had a 5 mm splenic artery aneurysm extending into the hilum. Since the gastroepiploic arteries create an anastomosis between the gastroduodenal and splenic artery [23], they could, in theory, compensate for decreased blood flow to the spleen in the case of celiac trunk stenosis that affects the splenic branch more than common hepatic flow resulting in a similar mechanism that leads to pancreaticoduodenal aneurysms. However, this is a hypothesis that would require further validation. Additionally, splenic artery aneurysms are the most common visceral artery aneurysm [24] and might, therefore, incidentally be present in a proportion of VAPUS patients. Even though we exclude splenic aneurysms in the currently proposed classification system, a subsequent expansion including them would be feasible as VAPUS X D.

The treatment outcomes were very favorable for these patients, especially in the case of elective surgery and intervention. In 14 patients that underwent invasive treatment, only a single patient did not survive the peri-operative period and the subsequent follow-up period after their index procedure. Considering the potential hazard of ruptured visceral artery aneurysms in the emergency setting, early elective treatment seems reasonable and justified. This is in accordance with previous findings [11,17,25,26].

A commonly discussed theory regarding the suspected pathomechanism behind IPDA aneurysms in the presence of upstream stenoses is the required increase in blood flow to compensate for the impaired end-organ supply leading to an increase in vessel diameter that can ultimately reach thresholds of aneurysmatic degradation. However, this hypothesis has not been conclusively proven so far and several factors contradict or at least question this theory. The IPDA is the first segment of the peri-pancreatic arcade that connects the SMA and celiac axis and therefore might be the first vessel exposed to increased blood flow from the SMA. However, IPDA aneurysms have been observed in cases of SMA stenosis/occlusion, which weakens the flow directional approach. Additionally, this hypothesis fails to produce an intuitive explanation for aneurysms of the gastroduodenal and superior pancreaticoduodenal arteries. As discussed above in the case of splenic aneurysms, there is a certain probability that flow obstructions of the celiac axis and SMA are simply coincidental. 

While the pathophysiology in classic Sutton–Kadir remaining to be conclusively investigated, the proposed classification system is not aimed at postulating a causal relationship between these pathologies. VAPUS rather offers an anatomic and clinical classification system that can be applied regardless of the causal relationship. Considering the potential number of combinations of visceral aneurysms and upstream flow impairment, of which many have been previously described in the respective literature, the proposed terminology might be able to improve the current state by means of standardization.

The visceral artery aneurysm in the presence of upstream stenosis (VAPUS) classification allows for a precise characterization of a previously broadly coined combination of pathologies. It enables both an accurate localization and indicates necessary treatment strategies by distinguishing between celiac trunk obstructions caused by MALC or atherosclerosis. Whether these pathologies are causally linked or coincidentally present at the same time is less relevant in clinical practice than developing a patient-specific treatment approach. Nevertheless, further studies are required to refine the proposed classification system especially when considering the rarity of potential obstruction/aneurysm combinations.

## Figures and Tables

**Table 1:** Baseline characteristics. Age and follow-up time are shown in years as median (first–third quartile) and refer to age at surgery or age at initial presentation in non-surgical cases.

	N = 16
*Female:Male*	8:8
*Surgical cases*	14 (87.5%)
*Age*	50 (47–62)
*Arterial hypertension*	10
Missing	3
*Smoking*	
Active	1
Past	2
Never	9
Missing	4
*30-day survival after surgery*	13 (92.9%)
*Follow-Up time*	3.4 (0.5–3.7)

**Table 2:** Proposed VAPUS classification system.

	Gastro-Duodenal Artery	Inferior Pancreatico-Duodenal Artery	Superior Pancreatico-Duodenal Artery
**Celiac Trunk** **Outside Compression**	1 A	1 B	1 C
**Celiac Trunk** **Endoluminal Occlusion**	2 A	2 B	2 C
**Superior Mesenteric Artery** **Endoluminal Occlusion**	3 A	3 B	3 C

**Table 3:** Aneurysmatic arteries and associated diameters. (CT = celiac trunk; SMA = superior mesenteric artery; MALC = median arcuate ligament compression; A = atherosclerosis; GDA = gastroduodenal artery; IPDA = inferior pancreaticoduodenal artery; SAPDA = superior anterior pancreaticoduodenal artery).

*Case*	*Stenosis*	*Etiology*	*Aneurysm*	*Proposed VAPUS Classification*	*Aneurysm Diameter (mm)*
*1*	CT	MALC + A	GDA	1–2 A	10.5
*2*	CT	MALC + A	IPDA	1–2 B	14.5
*3*	CT	MALC	IPDA	1 B	14
*4*	CT	MALC	IPDA	1 B	23.5
*5*	CT	MALC	IPDA	1 B	12.5
*6*	CT	MALC	IPDA	1 B	30
*7*	CT	MALC	IPDA	1 B	25
*8*	CT	MALC	IPDA	1 B	19
*9*	CT	MALC	IPDA	1 B	10
*10*	CT	MALC	SAPDA	1 C	26
*11*	CT	MALC	GDA + IPDA	1 A–B	21.5/12
*12*	CT	MALC + A	Splenic side branch	-	-
*13*	CT/SMA	MALC (CT) + A (SMA)	IPDA	1–3 B	15
*14*	CT	MALC	SAPDA	1 C	-
*15*	CT	MALC	IPDA	1 B	14
*16*	CT	MALC	GDA	1 A	12

**Table 4:** Summary of conducted treatments. (OSR = open surgical reconstruction; PTA = percutaneous transluminal angioplasty; MALC = median arcuate ligament compression; EEA = end-to-end anastomosis; CA = celiac artery).

*Case*	*OSR*	*Stent-PTA*	*Bypass*	*MALC*	*Embolization*
*1*	-	-	-	-	-
*2*	-	-	-	-	-
*3*	-	-	Aorto-hepatic	Release	Secondary intervention
*4*	Aneurysm resection (EEA)	-	-	Release	-
*5*	-	Secondary (CA)	-	Release	Secondary intervention
*6*	Aneurysm resection (EEA)	-	-	-	-
*7*	Aneurysm resection (EEA)	-	-	Release	-
*8*	-	-	-	Release	Secondary intervention
*9*	-	-	Aorto-hepatic	Release	Secondary intervention
*10*	Aneurysm resection (EEA)	-	-	Release	-
*11*	Aneurysm resection (EEA)	-	-	Release	-
*12*	Aneurysm ligation	-	-	-	-
*13*	-	Secondary (SMA)	Aorto-splenic	Release	Secondary intervention
*14*	-	-	Aorto-hepatic (secondary)	Release	During acute rupture
*15*	Aneurysm resection (EEA)	-	-	-	-
*16*	-	-	-	-	During acute rupture

## Data Availability

Data can be made available upon reasonable request to the corresponding author.
